# Relationships of internet gaming reasons to biological indicators and risk of internet gaming addiction in Korean adolescent male game users

**DOI:** 10.1186/s12888-020-02714-w

**Published:** 2020-06-30

**Authors:** Nahyun Kim, Mi Ja Kim, Tonda L. Hughes, Hyeweon Kwak, In Deok Kong

**Affiliations:** 1 Keimyung University College of Nursing, Daegu, South Korea; 2 University of Illinois at Chicago, College of Nursing, Chicago, IL USA; 3 Columbia University School of Nursing and Department of Psychiatry, Columbia University Irving Medical Center, New York City, NY USA; 4 Department of Nursing, Daekyeung University, Daegu, South Korea; 5 Department of Physiology, Yonsei University, Wonju College of Medicine, Wonju, Gangwon-Do 26426 Republic of Korea

**Keywords:** Addiction, Adolescent, Internet, Motivation, Norepinephrine

## Abstract

**Background:**

There are no standard diagnostic criteria or interventions for internet gaming addiction (IGA) even though IGA is one of the most pervasive public health issues among youth worldwide. Internet gaming reasons or motivations have been studied as a potential predictor of IGA, but the results have been inconsistent and biological indicators of gaming reasons have rarely been studied. We sought to (1) identify categories of internet gaming reasons, (2) examine the relationship of gaming reasons to risk of IGA, and (3) describe biological indicators associated with reasons for gaming.

**Methods:**

We used a multi-phase cross-sectional design including individual interviews; focus group discussion; and descriptive, comparative analysis. Fifteen Korean adolescent male internet gamers participated in individual interviews and eight participated in a focus group aimed at identifying reasons for internet gaming. Using the identified gaming reasons from these sources we surveyed 225 adolescent game users using a self-report questionnaire. Participants provided blood samples for assessment of norepinephrine (NE) and serum cortisol.

**Results:**

We identified four major categories of internet gaming reasons: entertainment, getting along with friends, stress relief, and habitual gaming. The habitual group showed significantly greater risk of IGA than the other groups (*p* < .001) and the lowest plasma NE levels (*p* = .035), possibly indicating an alteration in autonomic function.

**Conclusion:**

Health care providers are encouraged to screen adolescents for excessive internet gaming and to intervene with those who report habitual gaming behaviors. When feasible, assessment of biological indicators, such as plasma NE, may help to identify youth at greatest risk of IGA.

## Background

Excessive gaming behavior has received increasing attention in society and in academia and research over the past decade. In 2013, the American Psychiatric Association (APA) included excessive gaming behavior, designated as Internet Gaming Disorder (IGD), in the Fifth Edition of the Diagnostic and Statistical Manual of Mental Disorders (DSM-V) appendix as a potential mental disorder [1]. Furthermore, the World Health Organization (WHO) recently designated “Gaming Disorder” as a new diagnosis in the 11th final revision of the International Classification of Diseases (ICD-11) [2]. The diagnostic criteria for IGD and Gaming Disorder are similar because both were basically adapted from the DSM-V criteria for pathological gambling [1–3]. However, some controversy still exists about the inclusion of IGD in DSM-V and Gaming Disorder in ICD-11 due to lack of evidence [4–9]. Thus, more research is needed to identify clear criteria for diagnosis of IGD and/or Gaming Disorder. Regarding the terms applied to excessive gaming behavior, “addiction,” “disorder,” “pathological,” and “problematic” have been used interchangeably to describe individuals with this condition [10–12]. We primarily use the term “internet gaming addiction,” or IGA, in this paper because we believe that it is clearer and most commonly used in the research literature, including our prior studies [10–13]. IGA is defined as excessive or compulsive use of internet games that interferes with daily life; individuals with IGA tend to isolate themselves from social contact with others and to concentrate almost entirely on game activities [14].

As internet games have universally grown in popularity and IGA has become one of the most pervasive public health issues among youth worldwide [15], internet gaming motivations or reasons have been studied as one potential predictive risk factor for IGA. Understanding motivations or reasons for internet gaming is important because of their predictive and diagnostic value in identifying risk of IGA in internet game users. According to Deci and Ryan [16], game players should enjoy gaming insofar as it satisfies a player’s basic psychological need; thus, each player has different motives for playing games on the internet [17–19]. Furthermore, because game motivations can predict hours spent playing games [18, 19], understanding of individual differences in motives may be crucial to predict which gamers are at risk for pathological game use [20].

Yee [19] introduced 10 motivations within three major categories for online game playing: achievement, consisting of subcomponents of advancement, mechanics, and competition; social, consisting of socializing, relationship, and teamwork; immersion, consisting of discovery, role-playing, customization, and escapism. However, these ten motivations are diverse and often overlap [21], and the empirical results were inconsistent with regard to their relevance to risk of addiction [22]. For example, Caplan et al. [23] reported that immersion predicts problematic Internet use, whereas Zanetta Dauriat et al. [24] suggested that achievement, escapism, and socializing predict addictive gaming. In addition, Kuss et al. [22] indicated that escapism and mechanics significantly predict excessive gaming and proposed them as stronger predictors than time investment in gaming. Furthermore, other dimensions of gaming motivations have been introduced by several researchers [18, 21–23, 25–29]—many of which overlap with those suggested by Yee [19] and do not show consistent results regarding the association of IGA to gaming motivations or reasons [30].

Therefore, although many of the reasons are likely associated with internet gaming behavior, it remains unclear which reasons are most predictive of IGA [12, 13]. Furthermore, objective measures such as biological indicators have rarely been considered in relation to gaming reasons. Although autonomic functions have been found to be associated with internet gaming behaviors [31–37] few studies have examined their associations with gaming reasons.

Hence, our study aimed to fill this gap in the literature by examining reasons for internet gaming and the relationships of gaming reasons with IGA risk and with two biological indicators--plasma norepinephrine (NE) and serum cortisol--because these compounds reflect and are representative of autonomic responses to stimuli [38–40]. Although the terms “motivations” and “reasons” have been used interchangeably for internet gaming in previous studies, we use “internet gaming reasons” in this paper to refer to both motivations and reasons for excessive gaming.

## Methods

### Study design

This study is a multi-phase cross-sectional study that included individual interviews, focus group discussions, survey questionnaire, and biological analysis. It was a part of larger study that examined the role of the autonomic nervous system in development of IGA among adolescent males [36, 41, 42]. The current study focuses on identifying categories of gaming reasons and their relationships to IGA risk and to biological indicators (NE and cortisol levels). Although IGA risk and plasma NE and cortisol levels have been assessed in our previous studies, the research question and study sample are different. For example, the current study included only adolescents who reported being currently engaged in internet gaming.

### Participants and procedures

All study participants were male high school students (adolescents) who came to a regional health center in a city in South Korea in response to an advertisement about the study. Convenience and snowball sampling methods were used to recruit the sample. This study included three phases. In the first phase 15 adolescent males were interviewed individually; the second phase involved eight participants in a focus group discussion; and in the third phase 225 participants completed a questionnaire and provided a sample of blood.

Gaming reasons were generated in the first and the second phases of the study. Individual participants in the first phase were asked to describe gaming reasons. Participants in the second phase (focus group) were asked to affirm or revise/add to the gaming reasons obtained in the first phase. Both the individual interviews and the focus group interview were conducted in a private room, and responses were incorporated into the questionnaire used in phase three. Participants in the third phase first completed the questionnaire in a private room and then blood samples were drawn. All participants fasted for 12 h before blood sampling. In addition, participants were instructed not to smoke, drink caffeinated beverages, or engage in internet gaming for 24 h prior to data collection. This study was approved by the Institutional Review Board of a University. Informed consent was obtained from all participants and their legal guardians.

**Fig. 1:**
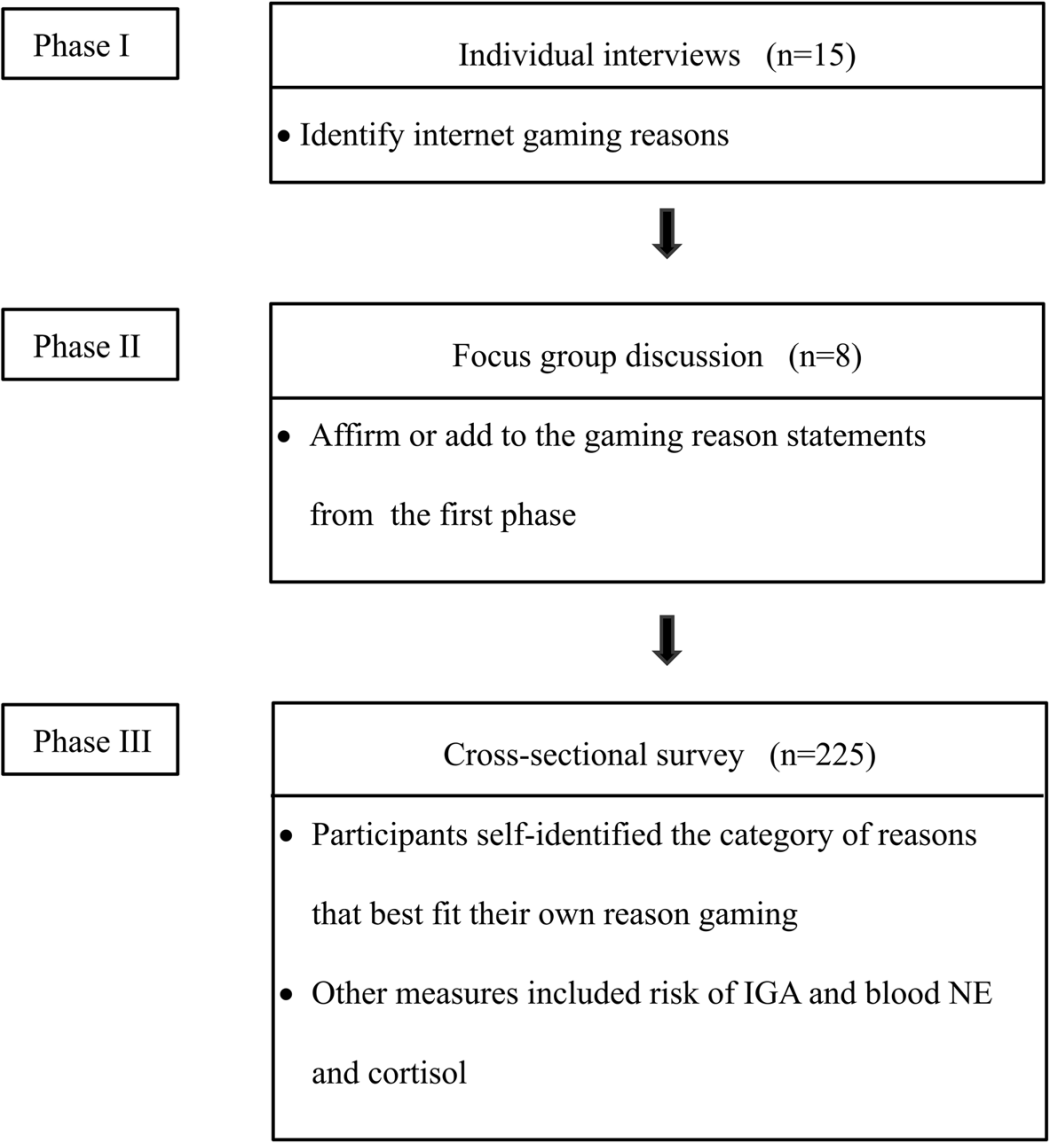
Flow chart of selection process of the study participants and data collection. IGA: internet gaming addiction, NE: norepinephrine

The sample size for the third phase was determined using one-way ANOVA analysis based on a medium effect size 0.25 [22, 24], an alpha level of 0.05, and a power of 0.80 using the G-power software [43]. A minimum sample size of 180 was estimated and we determined that and a sample size of 225 should provide ample power to detect statistically significant findings. The study sample was limited to male participants because male adolescents are known to be more commonly addicted to internet gaming than are their female counterparts [44]—and because reasons for gaming may differ by gender [19]. The flowchart for sampling procedures is depicted in Fig. 1.

### Variables

The variables considered for phase three of the study consisted of demographic characteristics; internet gaming-related information, including gaming reasons; IGA risk; and biological indicators. These variables, except biological indicators, were measured using a single questionnaire. The demographic characteristic- and internet gaming-related items (excluding gaming reasons) were generated from the literature; the content validity and reliability of the gaming reason-related items were established by four content experts. Biological indicators were assayed using blood samples.

#### Demographic characteristics

Demographic items included participant age as well as information about smoking, drinking, and sleep time. Data related to current smoking and to alcohol consumption were obtained using yes/no responses. Sleep time was obtained using two categories; six hours or more a day and less than six hours a day.

#### Internet gaming-related information

Internet gaming-related information included amount of time spent on internet gaming (minutes per day) and duration of internet gaming participation (years). Participants were asked to select one of four reasons that best described why they engaged in internet gaming. The categories included (1) entertainment, (2) getting along with friends (or friendship), (3) stress relief, and (4) habitual gaming. As described above, these categories were derived from individual interviews with 15 participants in the first phase and from the focus group interview with 8 participants in the second phase of the study. These interviews were performed by the principal investigator to identify perceived internet gaming reasons expressed in the adolescents’ own words. In the first phase, individual participants were asked to describe as many gaming reasons as possible in response to the question, “Why do you play internet games?” The duration of each interview ranged from 5 to 30 min. Overall, 36 statements were derived from the interview responses and were analyzed by two independent coders on the research team, both of whom were experienced in content analysis. In the second phase, statements derived in the first phase in were reviewed in a 45-min focus group interview in which 8 adolescents were asked about their agreement with the 36 statements and whether they could think of additional reasons for playing internet games. During the discussion participants were instructed to respond to questions by providing the first thought or feeling coming to their mind based on their own experiences and views. Participants’ responses were simple and brief, e.g., “because it’s fun,” “to play with friends,” or “just to do.” The 32 statements generated from the focus group discussion were similar to those generated in the first phase. A total of 68 statements were categorized by the two independent coders of the research team initially and then validated by participants in the focus group discussion.

#### Internet gaming addiction risk

IGA risk was measured using the IGA scale developed by the Korean Agency for Digital Opportunity and Promotion (KADO) [45]. The KADO scale was modified from Young’s Addictive Use of Internet scale [46] to assess the degree of IGA tendency in Korean adolescents. The KADO scale consists of three subscales that address game-oriented life, tolerance and loss of control, and withdrawal and affective experience [45]. This scale has been used to screen for IGA among Korean adolescents in annual national surveys. The IGA scale is a 20-item self-report measure; each item is rated on a 4-point Likert scale ranging from 1=“not at all” to 4 = “always.” The total score for the scale ranges from 20 to 80, with higher overall scores indicating greater risk of IGA. According to KADO, a scale score of 49 or above indicates high risk of IGA, and a score of 38 or above indicates overuse of games and potential risk of IGA. More details on the scale items are provided elsewhere [36]. The Cronbach’s alpha for the scale in the current study was 0.945, indicating high internal consistency.

#### Biological indicators

Peripheral venous blood samples for plasma NE and serum cortisol analyses were drawn from participants by professional nurses following standard laboratory procedures for assays. For each subject, 5 ml (mL) of venous blood was extracted using a heparin anticoagulation vacuum tube. Levels of plasma NE were analyzed using high-performance liquid chromatography (HPLC, Agilent 1200 series, Agilent Technology, USA). Serum cortisol levels were analyzed by chemiluminescent immunoassay using ADVIA Centaur and ADVIA Centaur XP systems (ADVIA Centaur XP, Siemens, USA). The ADVIA Centaur cortisol assay is a competitive immunoassay using direct chemiluminescent technology.

### Statistical analysis

Statistical analysis was performed to examine associations between reasons for gaming reasons and IGA risk and biological indicators. Data were analyzed using IBM SPSS statistics ver. 20.0 (IBM Co., Armonk, NY, USA). Descriptive statistics such as frequency, percentage, mean, and standard deviation were used to summarize the participants’ demographic and internet gaming-related characteristics. ANOVA was used to compare differences in levels of plasma NE and serum cortisol and IGA risk based on the four categories of internet gaming reasons, with Scheffe post-hoc tests. Analyses of categorical variables by the four gaming reason groups were analyzed using χ^2^-tests. A *p*-value of <.05 was considered statistically significant.

## Results

### Reasons for internet gaming

A total of 68 statements related to internet gaming reasons were grouped into four categories. Each category of reasons and related statements are summarized in Table 1. Among a total of 68 statements, 27 items (39.7%) were categorized as entertainment and included reasons such as “there are no leisure activities to do,” “playing games is fun,” and “eliminating boredom;” 17 items (25.0%) were categorized as friendship (e.g., “making friends,” “maintaining friendship,” and “enjoying gaming with friends”); 13 items (19.1%) were categorized as stress relief (e.g., “getting rid of stress,” “reducing tension,” and “relaxing”); 11 items (16.2%) were categorized as habitual gaming (e.g., “no other special reasons,” “habitually,” and “immersing myself in gaming regardless of my will”).

**Table 1:** Four internet gaming reasons in male adolescents

Categories	n (%)	Statement summary
Entertainment	27 (39.7)	. There are no leisure activities to do . Playing games is fun . Eliminating boredom . Passing time . Playing games as a hobby . Relieving boring
Friendship	17 (25.0)	. Making friends . Maintaining friendship . Enjoying gaming with friends . Being with friends. . Sharing consensus with friends . Bonding of sympathy with friends
Stress relief	13 (19.1)	. Getting rid of stress . Reducing tension . Relaxing . Forgetting something to do
Habitual gaming	11 (16.2)	. No other special reasons . Habitually . Immersing myself in gaming regardless of my will . Just to do so . Being addicted without noticing
Total	68 (100%)	

### Internet gaming reasons, risk of internet gaming addiction, and biological indicators

As shown in Table 2, the mean age of the 225 participants was 16.62 ± 1.03 years; mean age did not significantly differ among the internet gaming reason groups. Among the four groups, the habitual gaming group showed significantly greater IGA risk than the entertainment and friendship groups in terms of IGA scores (*F* = 9.120, *p* < .001). Daily internet gaming time was also significantly longer in the habitual gaming group than in the other groups (*F* = 4.958, *p* = .002). Plasma NE levels were lowest in the habitual gaming group among four gaming reason groups (*F* = 2.909, *p* = .035). Serum cortisol levels were slightly higher in the stress relief group than in the other groups, but the differences were not statistically significant (*F* = 0.606, *p* = .612). Duration of internet gaming and other individual behaviors such as smoking cigarettes, drinking alcohol, and sleep time did not significantly differ among the groups.

**Table 2:** Differences of internet gaming addiction risk and biological indicators according to the gaming reasons (*N* = 225)

		Internet gaming reasons					
		Entertainment^ⓐ^	Friendship^ⓑ^	Stress relief^ⓒ^	Habitual gaming^ⓓ^		
Variables						χ^2^/F	*p*
		(*n* = 65)	(*n* = 77)	(*n* = 30)	(*n* = 53)		
		n (%) / M ± SD	n (%) M ± SD	n (%) M ± SD	n (%) M ± SD		
Age (years)		16.60 ± 1.04	16.71 ± 1.04	16.50 ± 082	16.58 ± 1.12	0.377	.770
Smoking cigarettes	No (*n* = 167)	47 (28.1)	62 (37.1)	24 (14.4)	34 (20.4)	5.054	.168
	Yes (*n* = 58)	18 (31.0)	15 (25.9)	6 (10.3)	19 (32.8)		
Drinking alcohol	No (*n* = 169)	53 (31.4)	60 (35.5)	22 (13.0)	34 (20.1)	5.218	.156
	Yes (*n* = 56)	12 (21.4)	17 (30.4)	8 (14.3)	19 (33.9)		
Sleep time (hours per day)	≤ 6 (*n* = 147)	43 (29.3)	45 (30.6)	25 (17.0)	34 (23.1)	5.958	.114
	> 6 (*n* = 78)	22 (28.2)	32 (41.0)	5 (6.4)	19 (24.4)		
Internet gaming time (minutes per day)		162.92 ± 129.79	161.17 ± 123.26	139.67 ± 76.36	233.21 ± 150.66	4.958	.002^†^ ⓓ > ⓐⓑⓒ
Duration of internet gaming (years)		7.48 ± 2.56	7.14 ± 2.54	6.93 ± 2.21	7.38 ± 2.21	0.454	.715
IGA score		31.88 ± 11.17	36.21 ± 10.82	38.57 ± 13.29	42.85 ± 11.90	9.120	<.001^†^ ⓓ > ⓐⓑ
NE *(pg/ml)*		416.29 ± 280.69	391.78 ± 243.53	348.53 ± 195.26	297.02 ± 157.66	2.909	.035^†^ ⓓ < ⓐ
Cortisol *(μg/dl*)		2.24 ± 0.42	2.24 ± 0.41	2.36 ± 0.49	2.25 ± 0.49	0.606	.612

*Notes:* n = number, M: mean, SD: standard deviation, IGA: internet gaming addiction, NE: norepinephrine

^†^Scheffe test

## Discussion

In this study, we identified four major groups of internet gaming reasons: entertainment, getting along with friends (or friendship), stress relief, and habitual gaming. Furthermore, we investigated whether risk of IGA and biological indicators (i.e., NE and cortisol levels) differed by reasons for gaming in male adolescent game users. Entertainment, friendship, and stress relief were reported in earlier studies as common reasons for internet gaming [19, 21, 22, 47]. However, habitual gaming has not previously been reported as an internet gaming reason.

Our results partially support the hypothesis that IGA risk and biological indicators differ by gaming reason. Specifically, habitual game users showed significantly higher internet gaming time and IGA scores and lower NE levels than participants in the other gaming reason groups; however, no significant difference in cortisol levels was found. Instrumental behavior theory suggests that in contrast to goal-directed action, habitual control of action is characterized by involuntary, compulsive behavior [48, 49]. Habitual control of action is one important indicator of addiction [40], and therefore, a habitual gaming pattern may be an indicator of the level of addiction risk in heavy gamers. Our results seem to be consistent with those of earlier studies of alcohol abusers. In these studies, automatically activated motivations toward alcohol-related stimuli were associated with higher levels of addictive behaviors [50, 51].

A possible mechanism supporting the association between habitual gaming and IGA is explained by instrumental behavior theory. Based on this theory, behavior directed at obtaining rewards and avoiding punishment is controlled by a goal-directed system and a habit system [48]. Initial behavioral control is mainly goal-directed, but with repetition of an action, there is a gradual shift to habitual control [52]. During early engagement in internet gaming, adolescents may have specific goals or motivations for gaming such as entertainment, socialization, or escaping from reality. Goal-directed gaming activities seem to be initiated to achieve desirable outcomes and may be reinforced by the incentive value of the rewarding outcomes [53–55]. Over time, however, actions can become more and more habitual, and eventually they can be automatically evoked by triggering stimuli or motivations regardless of the possible outcomes [55, 56]. Some studies on addiction have shown that brain activity and structure are involved in changes from goal-directed behavior to habitual behavior [57–59]. Adolescents, in particular, are more likely than adults to develop habits because their executive inhibition skills are not yet fully developed [50, 51]. Under this line of reasoning, habitual gaming behavior would not be an initial motivation or reason for internet gaming but might be a consequence of gaming activities repeated for specific reasons. Schwabe and colleagues [40, 60–63] suggested that this transition from voluntary, goal-directed action to habitual control of action is stimulated by prolonged stress. There is strong evidence that stress and related hormones (e.g., glucocorticoid and norepinephrine) are important risk factors for development of a variety of addictions [40, 60, 61, 63, 64].

Notably, in our study, plasma NE levels were found to differ according to gaming reasons, which may support our assumption that the mechanism of habituation results from excessive internet gaming and stress-related physiological responses. Plasma NE levels were lower in the habitual gaming group than in the other groups; findings that are consistent with those of previous studies reporting associations of lower NE levels among internet addiction [37] and IGA [36] groups. These consistent findings in our study and in previous studies suggest that lower plasma NE levels in excessive internet gamers may be involved in the development of IGA, although the mechanisms of this involvement are unclear. In psychiatry research involving plasma NE, lower levels of NE were found in substance abusers than in healthy controls [65], and the mechanism was reported to be autonomic function dysregulation [11, 36, 65]. In contrast, serum cortisol levels did not significantly differ among the four groups in our study, a finding that was inconsistent with previous study results. In fact, a wealth of studies have reported higher serum cortisol levels in addicts, including substance abusers [41, 65, 66], due to HPA axis sensitization or blunting to stress [64, 65, 67]. However, some studies have also reported a negative association with cortisol levels, or no alterations in cortisol levels among people with pathological gambling or internet addiction [68, 69]. Interestingly, all these findings indicate alterations to HPA axis activity [67]. Our physiological results are the first reported with regard to internet gaming reasons or motivations. However, we are unable to explain the mechanisms of the phenomena observed, as this was not a focus of our study. Further research is needed to better understand the mechanisms underlying the relationship between autonomic function and gaming reasons.

Despite notable strengths of the study, the results should be evaluated within the context of some limitations. For example, we recognize that our study may not have included all reasons for internet gaming, nor did it assess game genre. Our use of a cross-sectional design limits interpretations of temporal order and because the sample was limited to male adolescents, we are unable to generalize findings to female adolescents or other population groups. Longitudinal studies with random samples are needed that include both male and female participants of various ages. In addition, studies conducted in countries other than Korea could shed light on how cultural contexts influence gaming behaviors and IGA.

## Conclusions

Our findings--that the habitual internet gaming group had the highest IGA risk and the lowest plasma NE levels--have potential implications for the clinical care of adolescent males. Health care workers can incorporate these findings in their health screenings of adolescent boys and initiate early preventive interventions with those who exhibit excessive internet game playing. Furthermore, researchers can use the information about reasons for engaging in internet gaming to further investigate the mechanisms underlying IGA. The study findings also have the potential to influence decisions about including IGA as a formal disorder in the DSM-V--a step that would encourage further research to develop gaming interventions for adolescents, regardless of where they live in the world.

## Data Availability

The datasets used and/or analyzed during the current study are available only upon reasonable request and after compliance with the policies and procedures of the Basic Science Research Program through the National Research Foundation of Korea and the Ministry of Education, Science and Technology (NRF) for data sharing.

## Abbreviations

DSM-V: Diagnostic and Statistical Manual of Mental Disorders, Fifth Edition
IGA: Internet Gaming Addiction
IGD: Internet Gaming Disorder
KADO: Korean Agency for Digital Opportunity and Promotion
NE: Norepinephrine
